# Chronic kidney disease and risk of atrial fibrillation and heart failure in general population‐based cohorts: the BiomarCaRE project

**DOI:** 10.1002/ehf2.13699

**Published:** 2021-11-26

**Authors:** Martin Rehm, Dietrich Rothenbacher, Licia Iacoviello, Simona Costanzo, Hugh Tunstall‐Pedoe, Catherine A. Fitton, Stefan Söderberg, Johan Hultdin, Veikko Salomaa, Pekka Jousilahti, Tarja Palosaari, Kari Kuulasmaa, Christoph Waldeyer, Renate B. Schnabel, Tanja Zeller, Stefan Blankenberg, Wolfgang Koenig, BiomarCaRE Consortium

**Affiliations:** ^1^ Institute of Epidemiology and Medical Biometry Ulm University Helmholtzstr. 22 Ulm D‐89081 Germany; ^2^ Department of Epidemiology and Prevention IRCCS Neuromed Pozzilli Italy; ^3^ Research Center in Epidemiology and Preventive Medicine (EPIMED). Department of Medicine and Surgery University of Insubria Varese Italy; ^4^ Cardiovascular Epidemiology Unit, Institute of Cardiovascular Research University of Dundee Dundee UK; ^5^ Division of Molecular and Clinical Medicine Dundee University Dundee UK; ^6^ Department of Public Health and Clinical Medicine Umeå University Umeå Sweden; ^7^ Department of Medical Biosciences, Clinical Chemistry Umeå University Umeå Sweden; ^8^ Finnish Institute for Health and Welfare Helsinki Finland; ^9^ Department of Cardiology University Heart and Vascular Center Hamburg Hamburg Germany; ^10^ German Center for Cardiovascular Research (DZHK e.V.), partner site Hamburg/Kiel/Lübeck Hamburg Germany; ^11^ Deutsches Herzzentrum München Technische Universität München Munich Germany; ^12^ DZHK (German Centre for Cardiovascular Research), partner site Munich Heart Alliance Munich Germany

**Keywords:** Cohort study, Chronic kidney disease, Atrial fibrillation, Heart failure, General population, Biomarkers

## Abstract

**Aims:**

Chronic kidney disease (CKD) has a complicated relationship with the heart, leading to many adverse outcomes. The aim of this study was to evaluate the relationship between CKD and the incidence of atrial fibrillation (AF) and heart failure (HF) along with mortality as a competing risk in general population cohorts. We also included an assessment of baseline biomarkers of inflammation, myocardial injury, and left ventricular dysfunction with risk of AF and HF, respectively, to shed light on the potential underlying pathophysiology.

**Methods and results:**

This study was conducted within the BiomarCaRE project using harmonized data from 12 European population‐based cohorts (*n* = 48 518 participants). Renal function was assessed by glomerular filtration rate estimated using the combined Chronic Kidney Disease Epidemiology Collaboration (CKD‐EPI) equation with standardized serum creatinine (Cr) and non‐standardized serum cystatin C (CysC). Incidence of AF and HF respectively, during a median follow‐up of 8 years was recorded. Cox proportional hazards models were used to determine hazard ratios (HRs) for the incidence of AF and HF in CKD and the competing risk of mortality after adjustment for covariates. The mean age at baseline was 51.4 (standard deviation 12.1) years, 49% were men. Overall, 4.3% of subjects had CKD at baseline. The rate for AF was 3.8 per 1000 person‐years during follow‐up. The HR for AF in patients with CKD compared with patients without CKD was 1.28 (95% confidence interval 1.07–1.54) after adjustment for covariates. The rate for incident HF was 4.1 per 1000 person‐years and the HR of CKD for HF was 1.71 (95% confidence interval 1.45–2.01. In subjects with CKD, N‐terminal‐pro‐brain natriuretic peptide (NT‐proBNP) showed an association with AF, whereas NT‐proBNP and C‐reactive protein were associated with HF.

**Conclusions:**

Chronic kidney disease is an independent risk factor for subsequent AF and is even more closely associated with HF. In these relatively young participants with CKD, NT‐proBNP was strongly associated with subsequent risk of AF. For HF, in addition, elevated levels of hs‐C‐reactive protein at baseline were related to incident events.

## Background

Chronic kidney disease (CKD) affects a large proportion of the adult population worldwide and is a global public health problem.^1^ Hypertension and diabetes are among the most important risk factors for CKD and account for much of the disease burden. CKD is also an independent risk factor for cardiovascular disease (CVD) and all‐cause mortality in both low‐risk and high‐risk cohorts.^2^

Chronic kidney disease has a complicated relationship with the heart, leading to many adverse consequences. Various pathophysiological interactions may occur, leading to haemodynamic and cardiac structural changes. These also predispose to atrial fibrillation (AF) and heart failure (HF).^3^ Both, AF^4^ and HF are associated with increased health care costs and mortality and, in combination with CKD, may complicate the disease course and worsen the risk for adverse outcomes.^5^ Given the ageing population and the increase in common risk factors for CKD, AF, and HF, such as hypertension, diabetes, and obesity, the prevalence of CKD and related heart disease will increase in the future. However, a better understanding of the shared pathophysiology, particularly in low‐risk populations, may help to further develop preventive strategies. Data from large population‐based initiatives with harmonized information and centrally measured biomarkers, and thus low study‐induced heterogeneity, may therefore be particularly valuable to investigate associations with sufficient power.

The aim of the study was to assess the relationship between CKD and the incidence of AF and HF along with mortality as a competing risk in participants of the MORGAM/BiomarCaRE consortium representing general population cohorts. We also included an assessment of established biomarkers of inflammation, myocardial injury, and left ventricular dysfunction to shed light on the potential underlying pathophysiology.

## Methods

### Study populations and study design

The present study was conducted as part of the MORGAM/BiomarCaRE projects with harmonized data from 12 population‐based cohorts from 4 European countries for which information on AF and HF during follow‐up was prospectively collected. Harmonized data included baseline information on age, sex, body mass index, smoking status, hypertension (defined as systolic blood pressure >140 mmHg, diastolic blood pressure >90 mmHg, or self‐reported use of blood pressure medication), and history of diabetes (defined as self‐report or antidiabetic medication). 48,518 subjects without a documented or self‐reported history of AF or HF at baseline and with complete information on baseline characteristics and laboratory measurements were included in the analysis. Details of the study cohorts are in *Supporting information Tables* **S1** and *S2*, *Box* **S1** and can be found elsewhere.^6^, ^7^, ^8^ All studies were carried out according to the Declaration of Helsinki. Each of the included contributing studies had previously obtained ethics approval from their respective institutional review boards, and all participants provided informed consent.

### Laboratory measurements

In the population‐based cohorts of the MORGAM/BiomarCaRE study, systolic blood pressure and total cholesterol were measured locally using standardized methods. Data quality was assessed retrospectively.^9^

Creatinine (Cr) was measured by the kinetic alkaline picrate Jaffe method using the isotope dilution mass spectrometry (IDMS) traceable (NIST SRM 967) Abbott Architect assay CREATININE on the Architect c8000. Cystatin C (CysC) was measured with the immunoassay Cystatin C on an Abbott Diagnostics ARCHITECT. All measurements were performed at the BiomarCaRE central laboratory in Mainz and, after the relocation of the lab, at the University Heart and Vascular Centre Hamburg‐Eppendorf in Hamburg.

Intra‐assay and inter‐assay coefficients of variation (CVs) were measured with samples of medium concentrations (creatinine: medium = 1.39–2.44 mg/dL; high = 2.25–7.3 mg/dL; cystatin C: high = 2.95–4.77 mg/L). Intra‐assay CVs for creatinine ranged from 0.09% to 5.2% and for cystatin C from 0.78% to 4.0%. The inter‐assay CV for creatinine ranged from 2.3% to 8.1% and for cystatin C from 1.8% to 12.5% for measurements in the population‐based cohorts.

Hs‐C‐reactive protein (CRP) was measured with a latex immunoassay on an Abbott Architect c8000 system. Troponin I levels were measured with an hs‐cTnI assay (Abbott Diagnostics; ARCHITECT i2000SR). N‐terminal‐pro‐brain natriuretic peptide (NT‐proBNP) was measured on the ELECSYS 2010 platform using an electrochemiluminescence immunoassay (ECLIA, Roche Diagnostics).

### Assessment of chronic kidney disease

Renal function was assessed by glomerular filtration rate (eGFR) estimated using the combined Chronic Kidney Disease Epidemiology Collaboration (CKD‐EPI) equation with standardized serum creatinine (Cr) and non‐standardized serum cystatin C (CysC).^10^

$$\text{eGFR}=\;177.6\times {\mathrm{Cr}}^{-0.65}\times {\text{CysC}}^{-0.57}\times {\mathrm{age}}^{-0.20}\times \left(0.82\;\text{if}\;\text{female}\right)\times \left(1.11\;\text{if}\;\text{black}\right)$$

In the equation, Cr is given in mg/dL, CysC in mg/L, age in years, and eGFR in mL/min/1.73 m^2^. CKD stage 3–5 was defined as eGFR of less than 60 mL/min/1.73 m^2^.

### Outcome definitions

The following outcomes were included in the analysis:

1. AF: Individuals with self‐reported and/or physician‐diagnosed history of AF/atrial flutter and/or prior coding for AF/atrial flutter at baseline were excluded from analyses. During follow‐up, the diagnosis of AF was based on study ECG tracings, questionnaire information, national hospital discharge registry data, including data on ambulatory visits to specialized hospitals. Additionally, causes of death registry data were screened for incident AF as a comorbidity of individuals that died from other causes. When routine clinical and death certificate diagnoses are used, the relevant International Classification of Diseases (ICD) codes are usually as follows: ICD‐8th Revision, 427.4; ICD‐9th Revision, 427.3; and ICD‐10th Revision, I48.
2. HF: Subjects with prevalent HF were also excluded from the analyses. For follow‐up assessment, each study centre was asked to decide on the exact definition of HF for their cohorts. The relevant ICD codes are usually as follows: ICD‐8th Revision, 427.0, 427.1, and 428; ICD‐9th Revision, 428; and ICD‐10th Revision, I50 (HF), I11.0 (hypertensive heart disease with HF), I13.0 (hypertensive heart and renal disease with HF), and I13.2 (hypertensive heart and renal disease with both HF and renal failure), which were adjusted according to local coding practices. In all cohorts, the follow‐up for HF was based on record linkage with hospital data and death registers and in FINRISK also with the national drug reimbursement register. The codes used, often based on national modifications of the ICD revisions, are specified in the MORGAM e‐publication.
3. Total mortality: Total mortality as an endpoint was defined as death due to any cause during the follow‐up time. More details of the event classification are provided elsewhere^7^, ^11^ and in the MORGAM manual.^8^ The follow‐up started at the date of baseline examinations. The duration of follow‐up in each cohort is described in *Table* **S1**.

### Statistical analysis

Baseline characteristics of the pooled study cohorts are presented as absolute and relative frequencies for categorical variables and means (standard deviations) or medians (25th, 75th percentiles) for continuous variables. Cumulative incidences and incidence rates per 1000 person‐years were calculated for AF, HF, and death as competing risks. Cumulative incidence curves were calculated using the Aalen–Johansen method.

The association of reduced renal function with AF, HF, and with death modelled as a competing risk was assessed using separate Cox proportional hazards regression models for each endpoint. Time since baseline was used as the time scale and eGFR category at baseline as the exposure (≥90, 60 to <90, and <60 mL/min/1.73 m^2^). The models were adjusted for age, sex, and cohort (Model 1) along with cardiovascular risk factors at baseline (body mass index, smoking status, diabetes, and systolic blood pressure) in Model 2. In a further analysis, an incremental adjustment for log‐transformed biomarker concentrations (hs‐CRP, hs‐cTnI, and NT‐proBNP) was performed based on Model 2. Non‐detectable biomarker measurements were set to half of the lower limit of detection of the assay. Subjects were censored at the end of follow‐up, upon death, or at the time of their cardiovascular event in the respective analyses. Scaled Schoenfeld residuals against follow‐up time were plotted for each covariate to verify the proportional hazards assumption.

Finally, in subjects with CKD, restricted cubic spline regression curves were used to visualize the association between eGFR and AF/HF and the association between log‐transformed biomarker concentrations and AF/HF. Statistical analysis was performed with SAS version 9.4 (SAS Institute, Cary, NC, USA) and R version 4.0.2 (R Foundation for Statistical Computing, Vienna, Austria).

## Results

Baseline characteristics of the 48 518 subjects included in the final analysis from 12 population‐based cohorts are shown in total and by eGFR category in *Table* *1*. The mean (standard deviation) age at baseline was 51.4 (12.1) years, and 49.0% were men. In the 2096 (4.3%) subjects with CKD Stage 3–5 (eGFR <60 mL/min/1.73 m^2^) at baseline, the prevalence of hypertension, age, and serum levels of biomarkers reflecting inflammation, myocardial injury, and left ventricular dysfunction (i.e. hs‐CRP, hs‐cTnI, and NT‐proBNP, respectively) were higher when compared to subjects with higher glomerular filtration rates. Further details on the included cohorts and their main baseline characteristics are presented in *Tables* *S1* and *S2*.

**Table 1:** Baseline characteristics of the study population by eGFR category

	eGFR, mL/min/1.73 m^2^			
	≥90 (*n* = 27 556)	60 to <90 (*n* = 18 866)	<60 (*n* = 2096)	Total (*n* = 48 518)
Age, years	46.7 (10.6)	56.8 (10.9)	64.6 (12.1)	51.4 (12.1)
0–44, *n* (%)	12 011 (43.6)	2588 (13.7)	139 (6.6)	14 738 (30.4)
45–54, *n* (%)	9129 (33.1)	5113 (27.1)	267 (12.7)	14 509 (29.9)
55–64, n (%)	5014 (18.2)	6434 (34.1)	546 (26.0)	11 994 (24.7)
65+, *n* (%)	1402 (5.1)	4731 (25.1)	1144 (54.6)	7277 (15.0)
Men, *n* (%)	14 613 (53.0)	8201 (43.5)	968 (46.2)	23 782 (49.0)
BMI, kg/m^2^	26.5 (4.5)	28.1 (4.8)	28.9 (5.1)	27.2 (4.7)
30+, *n* (%)	5146 (18.7)	5599 (29.7)	783 (37.4)	11 528 (23.8)
Smoking, *n* (%)	8660 (31.4)	4787 (25.4)	437 (20.8)	13 884 (28.6)
Hypertension, *n* (%)	9820 (35.6)	11 092 (58.8)	1608 (76.7)	22 520 (46.4)
History of diabetes, *n* (%)	863 (3.1)	1093 (5.8)	275 (13.1)	2231 (4.6)
SBP, mmHg	131.6 (18.7)	141.5 (21.4)	149.9 (23.8)	136.2 (20.8)
Total cholesterol, mmol/L	5.8 (1.2)	5.8 (1.2)	5.7 (1.3)	5.8 (1.2)
Creatinine, mg/dL	0.8 (0.1)	0.9 (0.2)	1.5 (0.9)	0.8 (0.3)
Cystatin C, mg/L	0.8 (0.1)	1.0 (0.1)	1.4 (0.5)	0.9 (0.2)
eGFR, mL/min/1.73 m^2^	108.4 (15.4)	78.6 (7.8)	49.9 (9.0)	94.3 (21.4)
hs‐CRP, mg/L	1.1 [0.5, 2.3]	1.7 [0.9, 3.5]	2.6 [1.3, 5.5]	1.4 [0.7, 2.9]
hs‐cTnI, ng/L	2.1 [1.3, 3.8]	2.8 [1.8, 4.5]	4.7 [3.0, 7.3]	2.5 [1.5, 4.3]
NT‐proBNP, ng/L	36.7 [17.9, 67.7]	56.3 [27.9, 106.2]	114.8 [54.3, 241.6]	44.8 [21.8, 85.7]

Baseline characteristics of the pooled study cohorts are presented as absolute and relative frequencies for categorical variables and means (standard deviations) or medians [25th, 75th percentiles] for continuous variables. BMI, body mass index; SBP, systolic blood pressure; eGFR, estimated glomerular filtration rate; hs‐CRP, high‐sensitivity C‐reactive protein; hs‐cTnI, high‐sensitivity cardiac troponin I; NT‐proBNP, N‐terminal pro B‐type natriuretic peptide.

The median follow‐up time was 8.0 years, with a mortality of 10.9% (5299 subjects died, mortality rate 10.0 per 1000 person‐years). As displayed in *Table* *2* 1999 (4.1%, rate 3.8 per 1000 person‐years) subjects were diagnosed with AF during the follow‐up period. In subjects with CKD, the HR for AF was 1.28 [95% confidence interval (CI) 1.07–1.54] after adjustment for covariates and decreased after biomarkers were included in the model (probable intermediate factors).

**Table 2:** Association of reduced kidney function with AF and death before AF as competing risk

	eGFR, mL/min/1.73m^2^			
	≥90 (*n* = 27 556)	60 to <90 (*n* = 18 866)	<60 (*n* = 2096)	Total (*n* = 48 518)
AF, *n* (%)	924 (3.4)	906 (4.8)	169 (8.1)	1999 (4.1)
IR (95% CI)	2.8 (2.6–2.9)	5.4 (5.1–5.8)	9.6 (8.3–11.2)	3.8 (3.7–4.0)
HR (95% CI)				
Model 1	1.00 (Reference)	1.18 (1.06–1.31)	1.44 (1.20–1.72)	
Model 2	1.00 (Reference)	1.10 (0.99–1.22)	1.28 (1.07–1.54)	
Model 3a	1.00 (Reference)	1.07 (0.97–1.19)	1.22 (1.02–1.47)	
Model 3b	1.00 (Reference)	1.03 (0.93–1.15)	1.10 (0.91–1.32)	
Model 3c	1.00 (Reference)	0.98 (0.88–1.09)	0.89 (0.74–1.07)	
Death before AF, *n* (%)	2416 (8.8)	1843 (9.8)	352 (16.8)	4611 (9.5)
IR (95% CI)	7.2 (6.9–7.5)	11.0 (10.5–11.5)	20.0 (18.0–22.2)	8.9 (8.6–9.1)
HR (95% CI)				
Model 1	1.00 (Reference)	1.09 (1.02–1.17)	1.56 (1.38–1.76)	
Model 2	1.00 (Reference)	1.07 (1.00–1.14)	1.45 (1.28–1.63)	
Model 3a	1.00 (Reference)	1.02 (0.96–1.10)	1.34 (1.18–1.51)	
Model 3b	1.00 (Reference)	1.00 (0.93–1.07)	1.25 (1.10–1.41)	
Model 3c	1.00 (Reference)	0.98 (0.92–1.05)	1.16 (1.03–1.32)	

AF, atrial fibrillation; IR, incidence rate per 1000 person‐years; HR, hazard ratio; CI, confidence interval.

Model 1: Adjusted for age, sex, and cohort. Model 2: Adjustment as in Model 1 plus adjustment for BMI, smoking, diabetes, and systolic blood pressure. Model 3a: Adjustment as in Model 2 plus adjustment for log‐transformed concentrations of hs‐CRP. Model 3b: Adjustment as in Model 2 plus adjustment for log‐transformed concentrations of hs‐CRP and hs‐cTnI. Model 3c: Adjustment as in Model 2 plus adjustment for log‐transformed concentrations of hs‐CRP, hs‐cTnI, and NT‐proBNP.

When both, CKD and HF at baseline, were assessed as variables in Model 2 (after HF at baseline was added to the data set only for these analyses), the HR for subsequent AF in the group with HF (*n* = 184) was 3.85 (95% CI 2.88–5.15) and the HR for subsequent AF in the presence of both HF and CKD (*n* = 35) was 3.50 (95% CI 1.95–6.29) (data not shown in tables). Unfortunately, a similar calculation was not possible for the combination of CKD with AF because no one had CKD and AF at baseline.

*Table* *3* displays the risk for HF with reduced kidney function. The HR was 1.71 (95% CI 1.45–2.01) after adjustment for covariates and only decreased slightly, after adjustment for hs‐CRP and thereafter hs‐cTnI. The C‐statistic for the Model 2 for incident AF was 0.81 (95% CI 0.80–0.82), whereas the C‐statistic for the Model 2 for incident HF was 0.84 (95% CI 0.83–0.85) (data not in tables).

**Table 3:** Association of reduced kidney function with HF and death before HF as competing risk

	eGFR, mL/min/1.73m^2^			
	≥90 (*n* = 27 556)	60‐ < 90 (*n* = 18 866)	<60 (*n* = 2096)	Total (*n* = 48 518)
HF, *n* (%)	806 (2.9)	1084 (5.7)	250 (11.9)	2140 (4.4)
IR (95% CI)	2.4 (2.2–2.6)	6.4 (6.1–6.8)	14.3 (12.6–16.1)	4.1 (3.9–4.3)
HR (95% CI)				
Model 1	1.00 (Reference)	1.41 (1.27–1.57)	2.07 (1.76–2.43)	
Model 2	1.00 (Reference)	1.27 (1.15–1.41)	1.71 (1.45–2.01)	
Model 3a	1.00 (Reference)	1.21 (1.09–1.34)	1.55 (1.31–1.82)	
Model 3b	1.00 (Reference)	1.16 (1.05–1.29)	1.36 (1.16–1.61)	
Death before HF, *n* (%)	2331 (8.5)	1683 (8.9)	304 (14.5)	4318 (8.9)
IR (95% CI)	6.9 (6.7–7.2)	10.0 (9.5–10.5)	17.3 (15.5–19.4)	8.3 (8.0–8.5)
HR (95% CI)				
Model 1	1.00 (Reference)	1.04 (0.97–1.12)	1.42 (1.25–1.61)	
Model 2	1.00 (Reference)	1.03 (0.96–1.10)	1.34 (1.18–1.52)	
Model 3a	1.00 (Reference)	0.99 (0.92–1.06)	1.25 (1.10–1.43)	
Model 3b	1.00 (Reference)	0.97 (0.90–1.04)	1.18 (1.03–1.34)	

HF, heart failure; IR, incidence rate per 1000 person‐years; HR, hazard ratio; CI, confidence interval.

Model 1: Adjusted for age, sex, and cohort. Model 2: Adjustment as in Model 1 plus adjustment for BMI, smoking, diabetes, and systolic blood pressure. Model 3a: Adjustment as in Model 2 plus adjustment for log‐transformed concentrations of hs‐CRP. Model 3b: Adjustment as in Model 2 plus adjustment for log‐transformed concentrations of hs‐CRP and hs‐cTnI.

**Figure 1:**
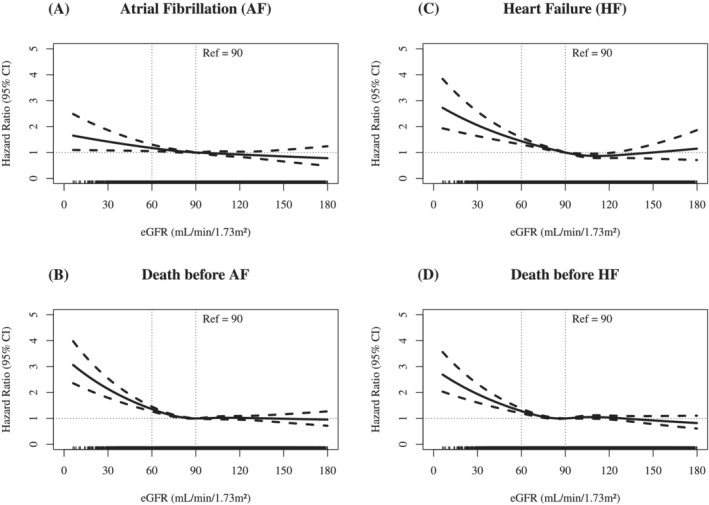
Restricted cubic spline regression curves [95% confidence interval (CI)] for the association between estimated glomerular filtration rate (eGFR) and atrial fibrillation (AF) or heart failure (HF) with death as competing risk adjusted for age, sex, cohort, body mass index (BMI), smoking, diabetes, and systolic blood pressure.

Restricted cubic spline regression curves for the association between eGFR and AF/HF incidence with death as a competing risk revealed a stronger association with a higher risk of HF than AF below an eGFR of 90 mL/min/1.73 m^2^, whereas no further risk change was observed above 90 mL/min/1.73 m^2^ (*Figure* *1*).

**Figure 2:**
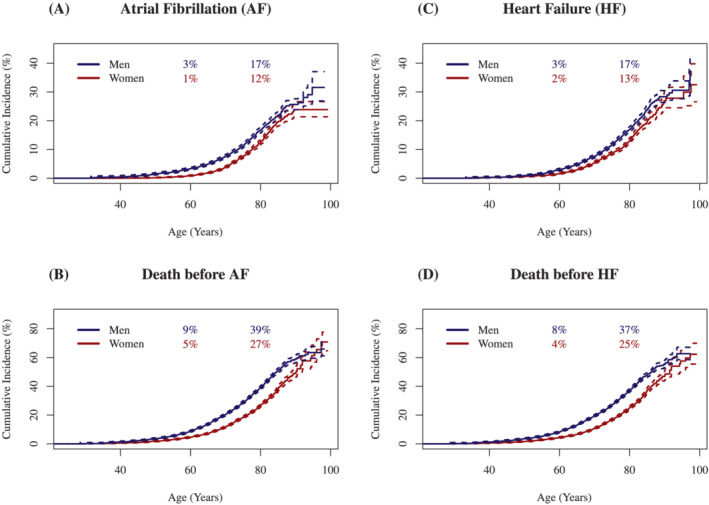
Cumulative incidence curves [95% confidence interval (CI)] for atrial fibrillation (AF) and heart failure (HF) with death as competing risk.

The cumulative incidence curves with death as a competing risk are shown in *Figure* *2*. A slightly higher incidence for men compared with women was evident for both AF and HF, although the curves for AF and HF did not differ significantly.

**Figure 3:**
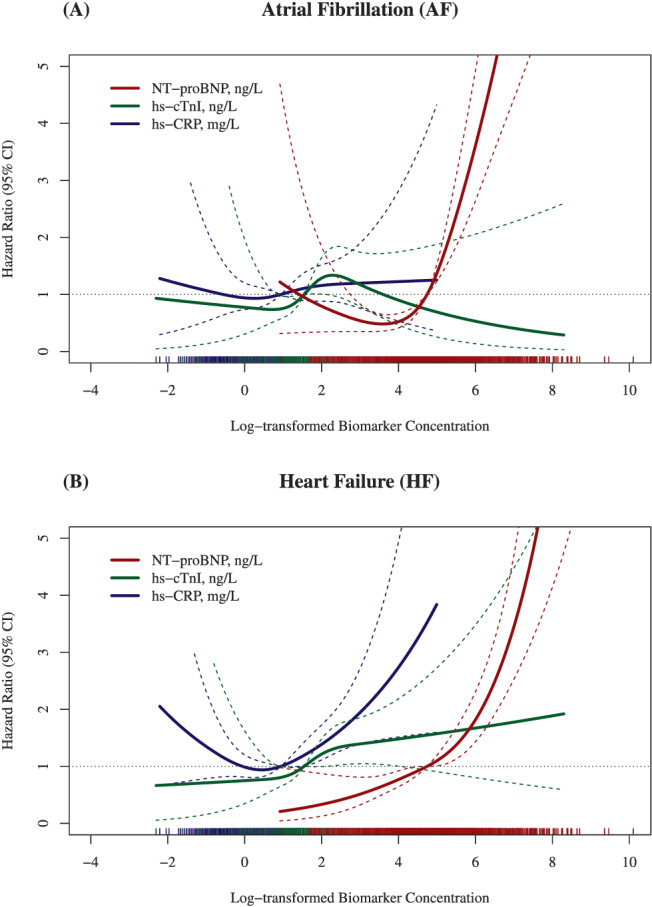
Restricted cubic spline regression curves [95% confidence interval (CI)] for the association between log‐transformed biomarker concentrations and atrial fibrillation (AF)/heart failure (HF) in subjects with chronic kidney disease (CKD) mutually adjusted for age, sex, cohort, body mass index (BMI), smoking, diabetes, and systolic blood pressure.

*Figure* *3* shows restricted cubic splines of the adjusted model in subjects with CKD (Model 2) when biomarkers were added to the model. Interestingly, only NT‐proBNP was associated with the risk of subsequent AF, whereas for the risk of HF, hs‐CRP was additionally predictive.

*Figure* *S1* shows the cumulative incidence of AF and HF in age categories and patients with CKD and diabetes. In particular, the risk of HF was significantly increased in patients with CKD as well as diabetes.

## Discussion

In this large prospective population‐based study conducted as part of the MORGAM/BiomarCaRE consortium, CKD was associated with the incidence of AF and even more strongly with HF, independent of other risk factors. The comorbidity of CKD and HF considerably increased the risk of AF. The study also suggested that in CKD, increased serum levels of NT‐proBNP, a well‐established biomarker for left ventricular dysfunction and haemodynamic stress, may play a role as an intermediate risk marker in the development of subsequent AF. Notably, in subjects with CKD, besides NT‐proBNP, inflammation as assessed by hs‐CRP might also play a role in the risk of subsequent HF.

### Incidence of atrial fibrillation and heart failure in general population‐based studies

Atrial fibrillation is the most common arrhythmia in adults and the estimated prevalence in the general population aged 20 years and older is about 3%.^11^, ^12^ Chronic conditions such as hypertension, obesity, coronary heart disease, HF, and diabetes further increase the risk of AF. The burden of AF appears to be lower in women than in men, but mortality is similar, a finding supported by our data. A detailed sex‐specific analysis within the BiomarCaRE consortium suggests that the observed differences in AF incidence may be explained by the sex‐specific distribution of risk factors.^13^

The prevalence of HF in the adult population in developed countries is about 1–2%, rising to over 10% in people aged 70 years or older.^14^ The underlying cause is multifactorial, but many patients with HF have a history of myocardial infarction or coronary heart disease.

### The cardio‐renal syndrome: pathophysiological and clinical consequences

Subjects with CKD are already at increased risk for non‐fatal CVD events and death.^2^ Not only do CKD and AF share many common risk factors, such as older age, obesity, hypertension, diabetes, and smoking,^15^ there are also specific effects of CKD on cardiac structure, endothelial function, and vascular calcification.^17^ The chronic fluid overload and increased afterload due to arterial stiffness can lead to left ventricular hypertrophy with marked myocardial fibrosis, and consequently to left ventricular diastolic dysfunction and left atrial enlargement.^17^ In particular, chronic interactions (so‐called type 4 subtype) of cardio‐renal syndromes may further be characterized by haemodynamic changes due to low cardiac output and altered venous return. In addition, neurohormonal mechanisms triggering the renin–angiotensin–aldosterone system (RAAS) leading to further fluid overload, inflammatory changes, renal and cardiac fibrotic remodelling processes, and finally organ degeneration play an important role.^16^

As seen in our general low‐risk study population, elevated levels of a marker of left ventricular dysfunction and haemodynamic stress (NT‐proBNP) at baseline were associated with the development of AF in subjects with CKD, whereas markers of inflammation (hs‐CRP) and myocardial damage (hs‐cTnI) were not. While only left ventricular dysfunction was associated with future risk of AF, the pattern was different in HF, for which hs‐CRP and NT‐proBNP were both strong predictors. These differences most likely reflect differences in cardiovascular pathology rather than impaired renal clearance.^17^

In addition, diabetes is an important risk factor especially for HF, as can also be seen in our data.

### Implications

From a public health perspective, treatment strategies of early stages of a cardio‐renal syndrome include preventive measures and management of risk factors and concomitant diseases.^18^ Lifestyle changes such as weight reduction, adequate control of blood pressure and atherogenic lipoproteins, glycaemic status, and smoking cessation are crucial elements of an early treatment strategy.^16^ In addition, patients with CKD and AF are at particular risk for stroke and need oral anticoagulation. Therefore, stroke bleeding risk reassessment at periodic intervals is recommended.^14^, ^18^ However, because the population with CKD, AF, and especially HF will increase in the near future, more studies are needed to inform a decision about beneficial therapies in the early stages of these comorbid diseases.

### Strengths and limitations

We included a large number of general population‐based studies, used harmonized data, and could rely on centralized measurements for creatinine and cystatin C. We also used creatinine and cystatin C in combination, to define glomerular filtration as it strengthened the association between CKD and outcomes.^19^ Limitations include that we relied on one single measurement only to define CKD. Unfortunately, we also could not include measurements of albumin in urine as it was not available in a standardized manner in all included studies. As CKD and the heart have a bidirectional relationship, increased levels of a cardiac biomarker such as NT‐proBNP might also be a consequence of CKD. To minimize this potential source of bias and to assure that exposure preceded the new occurrence of AF, or respectively HF, subjects with prevalent AF or HF at baseline were excluded from the analyses. In addition, the association between biomarker concentrations and AF/HF (as displayed in *Figure* *3*) was only analysed in subjects with CKD. Notably, other adverse sequelae of the cardio‐renal syndrome such as anaemia and bone and mineral disorders (e.g. osteoporosis) have to be considered also but had not been investigated in the context of this study.

## Conclusions

Chronic kidney disease is a risk factor for the subsequent occurrence of AF and even more so for HF. The risk for AF is particularly high when CKD and HF are comorbid conditions. In these general relatively young and low‐risk populations, only left ventricular dysfunction/myocardial stress together with CKD was associated with the occurrence of AF, even after adjustment for covariates. However, concerning the risk of subsequent HF, inflammation, as reflected by increased serum levels of hs‐CRP, played a significant role in addition to NT‐proBNP.

## Conflict of interest

All authors have completed the ICMJE uniform disclosure form online (www.icmje.org/coi_disclosure.pdf) and declare no support from any organization for the submitted work; no financial relationships with any organizations that might have an interest in the submitted work in the previous three years; no other relationships or activities that could appear to have influenced the submitted work.

Outside the submitted work, S.B. reports personal fees from Lumira Diagnostics, ThermoFisher, Siemens, Abbott Diagnostics, Roche Diagnostics, and grants from ThermoFisher, Siemens, and Abbott Diagnostics. S.S. reports personal fees from Actelion Ltd.; V.S. reports personal fees from Novo Nordisk, Sanofi, and grants from Bayer AG. W.K. reports personal fees from AstraZeneca, Novartis, Pfizer, The Medicines Company, DalCor, Kowa, Amgen, Corvidia, Berlin‐Chemie, DalCor, Genentech, Sanofi, Daichii‐Sankyo, Esperion, OMEICOS, LIB Therapeutics and Novo Nordisk in addition to grants and non‐financial support from Beckmann, Singulex, Abbott, Roche Diagnostics, all outside the scope of the present paper. All other authors declare no conflict of interest.

## Funding

This work was supported by the 7th Framework Programme Collaborative Project (grant agreement no. HEALTH‐F2‐2011‐278913). The MORGAM Project has received funding from EU projects MORGAM (Biomed, BMH4‐CT98‐3183), GenomEUtwin (Fifth Framework Programme FP5, QLG2‐CT‐2002‐01254), ENGAGE (FP7, HEALTH‐F4‐2007‐201413), CHANCES (FP7, HEALTH‐F3‐2010‐242244), BiomarCaRE (FP7, HEALTH‐F2‐2011‐278913), euCanSHare (Horizon 2020, No. 825903) and AFFECT‐EU (Horizon 2020, No. 847770); and Medical Research Council, London (G0601463, No. 80983: Biomarkers in the MORGAM Populations). This has supported central coordination, workshops, and part of the activities of the MORGAM Data Centre, the MORGAM Laboratories and the MORGAM Participating Centres, and the MORGAM Biomarker Laboratory at Johannes Gutenberg University in Mainz, Germany. MORGAM Participating Centres are funded by regional and national governments, research councils, charities, and other local sources. Dr Zeller was supported by the German Center of Cardiovascular Research (DZHK e.V.) under Grant number 81Z1710101. Stefan Blankenberg reports grants from Abbott Diagnostics, during the conduct of the study. Dr Salomaa was supported by the Finnish Foundation for Cardiovascular Research.

## Supporting information


**Table S1.** List of included population‐based cohorts (Complete Set).
**Table S2.** Main characteristics of included subjects by cohort (Final Analysis Set).
**Figure S1.** Cumulative incidence (95% CI) of AF and HF by absence or presence of CKD and diabetes by age group at baseline.
**Box S1.** Further Description of Study Cohorts.
