# Supplementary material for: Chronic kidney disease and risk of atrial fibrillation and heart failure in general population‐based cohorts: the BiomarCaRE project
Source: ESC Heart Fail. 2021 Nov 26;9(1):57–65. doi: 10.1002/ehf2.13699 (PMC8788046; doi:10.1002/ehf2.13699)
Supplement: Supplementary file 1 — Table S1. List of included population‐based cohorts (Complete Set). Table S2. Main characteristics of included subjects by cohort (Final Analysis Set). Figure S1. Cumulative incidence (95% CI) of AF and HF by absence or presence of CKD and diabetes by age group at baseline. Box S1. Further Description of Study Cohorts. [file EHF2-9-57-s001.docx]

**Supplementary Table S1** List of included population-based cohorts (Complete Set)

| **Cohort** | **Country** | **n** | **Baseline Assessment** | **Median Follow-up,**  **years** | **Creatinine measurement,**  **n (%)** | **Cystatin C measurement,**  **n (%)** | **hs-cTnI measurement,**  **n (%)** | **hs-CRP measurement,**  **n (%)** | **NT-proBNP measurement,**  **n (%)** |
| --- | --- | --- | --- | --- | --- | --- | --- | --- | --- |
| FINRISK 97 | Finland | 8444 | 1997 | 13.8 | 7872 (93.2) | 7900 (93.6) | 7230 (85.6) | 7954 (94.2) | 7832 (92.8) |
| Moli-sani | Italy | 24325 | 2005-2010 | 4.3 | 23647 (97.2) | 23666 (97.3) | 23483 (96.5) | 23604 (97.0) | 23696 (97.4) |
| N-Sweden 86 | Sweden | 1625 | 1986 | 25.8 | 1601 (98.5) | 1601 (98.5) | 1575 (96.9) | 1601 (98.5) | 1599 (98.4) |
| N-Sweden 90 | Sweden | 1576 | 1990 | 21.8 | 1421 (90.2) | 1421 (90.2) | 1370 (86.9) | 1420 (90.1) | 1423 (90.3) |
| N-Sweden 94 | Sweden | 1893 | 1994 | 17.9 | 1861 (98.3) | 1854 (97.9) | 1789 (94.5) | 1859 (98.2) | 1860 (98.3) |
| N-Sweden 99 | Sweden | 1789 | 1999 | 12.8 | 1726 (96.5) | 1725 (96.4) | 1697 (94.9) | 1723 (96.3) | 1720 (96.1) |
| N-Sweden 04 | Sweden | 1863 | 2004 | 7.8 | 1810 (97.2) | 1810 (97.2) | 1809 (97.1) | 1810 (97.2) | 1799 (96.6) |
| N-Sweden 09 | Sweden | 1704 | 2009 | 2.8 | 1627 (95.5) | 1627 (95.5) | 1538 (90.3) | 1627 (95.5) | 1619 (95.0) |
| SHHEC 1 | UK | 11573 | 1984-1987 | 23.4 | 10776 (93.1) | 10781 (93.2) | 9607 (83.0) | 10763 (93.0) | 8930 (77.2) |
| SHHEC 21 | UK | 1016 | 1989 | 20.7 | 680 (66.9) | 680 (66.9) | 201 (19.8) | 674 (66.3) | 181 (17.8) |
| SHHEC 2 | UK | 1754 | 1992 | 17.5 | 1651 (94.1) | 1651 (94.1) | 1394 (79.5) | 1650 (94.1) | 1451 (82.7) |
| SHHEC 3 | UK | 1656 | 1995 | 14.5 | 1640 (99.0) | 1640 (99.0) | 1422 (85.9) | 1640 (99.0) | 1448 (87.4) |
| Overall |  | 59218 |  | 8.0 | 56312 (95.1) | 56356 (95.2) | 53115 (89.7) | 56325 (95.1) | 53558 (90.4) |

hs-cTnI, high-sensitivity cardiac troponin I; hs-CRP, high-sensitivity C-reactive protein; NT-proBNP, N-terminal pro B-type natriuretic peptide; SHHEC, Scottish Heart Health Extended Cohort.

**Supplementary Table S2** Main characteristics of included subjects by cohort (Final Analysis Set)

| **Cohort** | **Country** | **n** | **Age (years),**  **mean (SD)** | **Men,**  **n (%)** | **Diabetes,**  **n (%)** | **eGFR (ml/min/1.73m²),**  **mean (SD)** | **Incident AF,**  **n (%)** | **Incident HF,**  **n (%)** |
| --- | --- | --- | --- | --- | --- | --- | --- | --- |
| FINRISK 97 | Finland | 6518 | 47.5 (13.2) | 3217 (49.4) | 344 (5.3) | 91.6 (18.2) | 286 (4.4) | 416 (6.4) |
| Moli-sani | Italy | 21496 | 54.9 (11.7) | 10236 (47.6) | 1342 (6.2) | 87.5 (17.2) | 315 (1.5) | 504 (2.3) |
| N-Sweden 86 | Sweden | 1546 | 45.4 (11.2) | 801 (51.8) | 50 (3.2) | 110.5 (22.1) | 154 (10.0) | 114 (7.4) |
| N-Sweden 90 | Sweden | 1353 | 44.7 (11.2) | 677 (50.0) | 31 (2.3) | 103.9 (20.4) | 98 (7.2) | 79 (5.8) |
| N-Sweden 94 | Sweden | 1739 | 49.7 (13.9) | 880 (50.6) | 60 (3.5) | 91.9 (22.3) | 147 (8.5) | 118 (6.8) |
| N-Sweden 99 | Sweden | 1619 | 50.0 (13.9) | 807 (49.8) | 58 (3.6) | 93.2 (24.7) | 94 (5.8) | 66 (4.1) |
| N-Sweden 04 | Sweden | 1603 | 50.6 (14.2) | 774 (48.3) | 83 (5.2) | 118.6 (28.8) | 63 (3.9) | 39 (2.4) |
| N-Sweden 09 | Sweden | 1436 | 50.6 (13.8) | 744 (51.8) | 72 (5.0) | 102.2 (26.1) | 9 (0.6) | 3 (0.2) |
| SHHEC 1 | UK | 8515 | 49.1 (7.3) | 4283 (50.3) | 126 (1.5) | 101.7 (21.2) | 649 (7.6) | 617 (7.2) |
| SHHEC 21 | UK | 137 | 49.5 (10.2) | 53 (38.7) | 5 (3.6) | 104.3 (18.5) | 14 (10.2) | 9 (6.6) |
| SHHEC 2 | UK | 1274 | 53.3 (13.9) | 642 (50.4) | 28 (2.2) | 97.6 (22.6) | 114 (8.9) | 130 (10.2) |
| SHHEC 3 | UK | 1282 | 46.1 (11.6) | 668 (52.1) | 32 (2.5) | 103.6 (18.9) | 56 (4.4) | 45 (3.5) |
| Overall |  | 48518 | 51.4 (12.1) | 23782 (49.0) | 2231 (4.6) | 94.3 (21.4) | 1999 (4.1) | 2140 (4.4) |

eGFR, estimated glomerular filtration rate; AF, Atrial Fibrillation; HF, Heart Failure; SHHEC, Scottish Heart Health Extended Cohort.

**Supplementary Figure S1** Cumulative incidence (95% CI) of AF and HF by absence or presence of CKD and diabetes by age group at baseline.


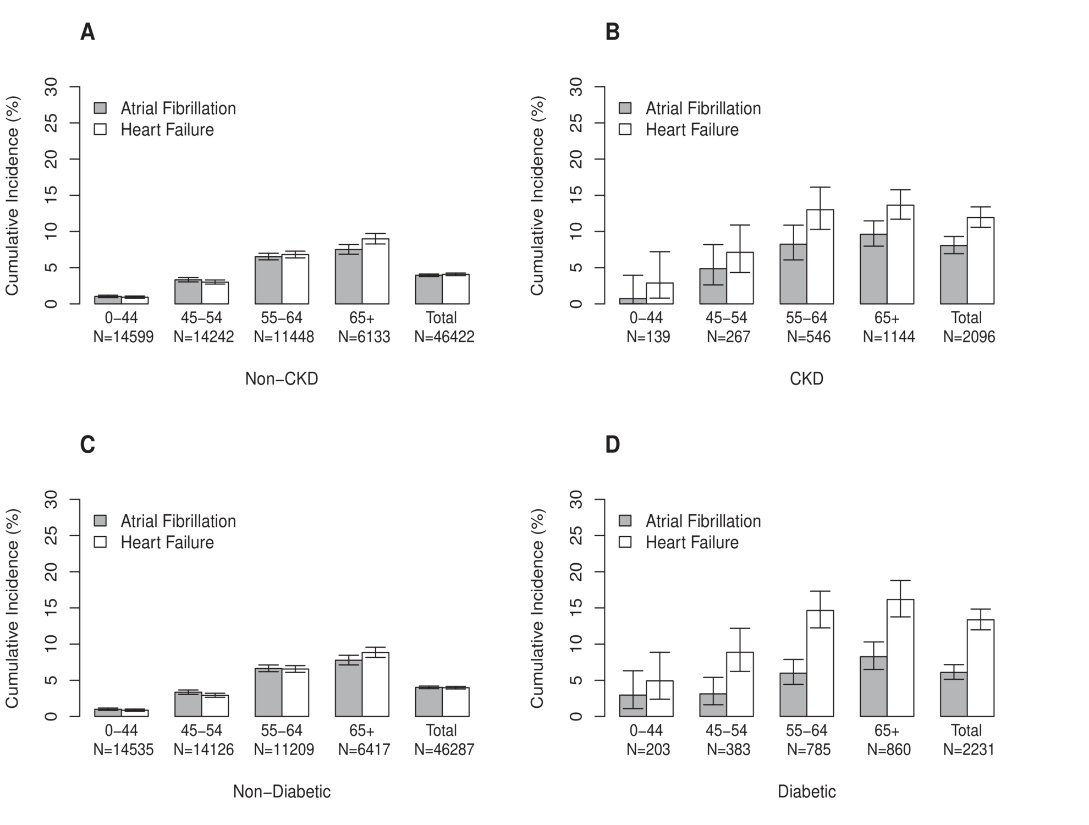


**Box S1** Further Description of Study Cohorts

| **General Population Based Cohorts** |
| --- |
| **FINRISK:** The FINRISK study is a series of population-based cardiovascular risk factor surveys carried out every five years in five (or six in 2002) districts of Finland, including North Karelia, Northern Savo (former Kuopio), Southwestern Finland, Oulu Province, Lapland province (in 2002 only) and the region of Helsinki and Vantaa. A stratified random sample was drawn for each survey from the national population register, the age-range was 25-74 years. All individuals enrolled in the study received a physical examination, a self-administered questionnaire, and a blood sample was drawn. In 1997, altogether 11,500 individuals were invited and 8,444 (73%) participated in the clinical examination. During follow-up the National Hospital Discharge Register, the National Causes of Death Register and the National Drug Reimbursement Register were used to identify endpoints. At the moment, the follow-up extends until Dec. 31^st^, 2010, i.e., 14 years for the FINRISK 1997 cohort. The Coordinating Ethics Committee of the Helsinki and Uusimaa Hospital District approved the study, which followed the declaration of Helsinki. All subjects gave written informed consent. <http://www.thl.fi/publications/morgam/cohorts/full/finland/fin-fina.htm> |
| **Moli-sani Project:** The cohort of the Moli-sani Project was recruited in the Molise region from city hall registries by a multistage sampling. First, townships were sampled in major areas by cluster sampling; then, within each township, participants aged 35 years or over were selected by simple random sampling. Exclusion criteria were pregnancy at the time of recruitment, lack of in understanding, current multiple trauma or coma, or refusal to sign the informed consent. A total of 24,325 men (47%) and women (53%) over the age of 35 were examined at baseline from 2005 to 2010. Participation was 70%. The cohort was followed-up for a median of 4.2 years (maximum 6.5 years) at December 2011. Follow-up and assessment of AF and HF events was achieved by linking records to hospital discharge registers and national cause of death registers. Validation of events was achieved by linking hospital records and physician records using updated MORGAM criteria. <http://www.moli-sani.org/>  <http://www.thl.fi/publications/morgam/cohorts/full/italy/ita-mola.htm> |
| **The Northern Sweden MONICA Study:** The Northern Sweden MONICA study covered the two northernmost counties of Sweden, i.e. Norrbotten and Västerbotten with altogether 510,000 inhabitants. Population surveys were performed in 1986, 1990, 1994, 1999, 2004 and 2009, with altogether 10,517 unique participants.^18^ On the first two occasions, 2,000 persons aged 25 to 64 years were randomly selected, and in the last three surveys, the upper age limit was extended to 74 years and 2,500 individuals were invited. A stratified randomized selection procedure by age and sex (250 persons in each sex/10-year age stratum) has been used. The participation rate was 69-81%. Detailed analyses of non-participants have been performed. Incident cardiovascular events (myocardial infarction and stroke) occurring in the region between 1985 and 2010 and below the age of 75 were collected and validated according to MONICA criteria by two event registers whose accuracy and validity have been tested against national registers. Follow-up is available for all cohorts until December 2011 for mortality and non- fatal coronary, stroke, chronic heart failure, atrial fibrillation, cancer and diabetes events. Coronary and stroke events below the age of 75 validated applying the MONICA diagnostic criteria, and diabetes according to careful case review. , <http://www.thl.fi/publications/morgam/cohorts/full/sweden/swe-nswa.htm> |
| **Scottish Heart Health Extended Cohort (SHHEC):** This consists of two overlapping studies which share a common protocol and methods: the Scottish Heart Health Study randomly recruited men and women aged 40-59 across 22 Scottish districts in 1984-1987; Scottish MONICA similarly recruited men and women aged 25-64 in Edinburgh and North Glasgow in 1986, and in North Glasgow again in 1989, 1992 (to 74), and in 1995 as part of the WHO MONICA Project. They are now combined as one cohort, although length of follow-up, currently to the end of 2009, inevitably varies in the different components. Follow up is achieved through flagging for death certificates at the National Health Service Death Register and through the Scottish Record Linkage scheme for deaths and hospital discharge records run by Information Services Scotland, which works on probability matching. These diagnoses are no longer validated from case notes now that endpoint numbers run into thousands as the cohort ages, but they are allocated to MORGAM categories. Risk factor and endpoint data were used to produce the ASSIGN cardiovascular risk score.^23^ See. <http://www.thl.fi/publications/morgam/cohorts/full/uk/unk-sco.htm> |
